# Editorial: Hepatocellular carcinoma: novel treatment strategies, volume III

**DOI:** 10.3389/fmed.2025.1690673

**Published:** 2025-09-23

**Authors:** Fan Feng, Xiaojie Xu

**Affiliations:** ^1^Clinical Laboratory, The Fifth Medical Center of Chinese People's Liberation Army General Hospital, Beijing, China; ^2^Department of Genetic Engineering, Beijing Institute of Biotechnology, Beijing, China

**Keywords:** HCC, liver cancer, therapeutic strategies, TKIs, ICIs

Hepatocellular carcinoma (HCC) is the most common pathological type of liver tumor, and its risk factors vary significantly across countries or regions (1). In Europe and the United States, alcoholic liver disease predominates, whereas in East Asia-Pacific regions represented by mainland China, HBV, HCV, and other hepatitis viruses, along with virus-related acute and chronic liver diseases, are the primary contributors (2). Despite substantial progress in antiviral treatments for HBV and HCV, as well as the significant impact of mandatory universal HBV vaccination for newborns, mainland China still has over 80 million people infected with HBV or HCV or suffering from virus-related acute and chronic liver diseases (2, 3). Consequently, HBV-related research remains critically important (4). Our Research Topic “Hepatocellular Carcinoma: Novel Treatment Strategies” focuses on new advances and strategies in HCC treatment and has now reached its third volume. Through this editorial, we aim not only to introduce the articles published in this Research Topic but also to encourage the submission of more related research works for the forthcoming “Volume IV.”

The current landscape of HCC treatment has been revolutionized by immune checkpoint inhibitors (ICIs), targeted therapies, and novel therapeutic combinations. Yang et al. summarize pivotal findings from the 2024 ASCO Annual Meeting, highlighting emerging therapies including ICIs, CAR-T cell therapies, oncolytic viruses, and locoregional treatments such as transarterial chemoembolization (TACE) and hepatic arterial infusion chemotherapy (HAIC), thereby underscoring the imperative for sustained innovation. Future efforts should prioritize overcoming resistance mechanisms, optimizing combination regimens, and integrating biomarker-driven approaches to enhance clinical outcomes and advance personalized treatment paradigms.

Malignancies such as hepatocellular carcinoma (HCC) represent complex, polygenic diseases, making multi-gene, and multi-factor panel-based diagnostic models a current research trend. For instance, Shi and Hu established a machine learning-based prediction model to assess the prognosis of male HBV-induced HCC patients with smoking and alcohol consumption habits following local ablation treatment. This model identifies key risk factors, including monocyte levels, globulin concentrations, and the platelet-albumin-bilirubin (PALB) score, providing accurate prognostic predictions for this specific patient subgroup. Separately, Liu et al. employed bioinformatics analysis to construct a prognostic framework incorporating six critical genes: ANXA2, APOA1, EZH2, IGF2BP3, SQSTM1, and TNFRSF11B.

Concurrently, the landscape of HCC treatment strategies has evolved from monotherapy to multimodal combinations, progressing from dual-therapy regimens to triple or even quadruple therapeutic approaches. Zhang et al. demonstrated the prognostic significance of early alpha-fetoprotein (AFP) and des-gamma carboxy prothrombin (DCP) responses in unresectable HCC patients undergoing triple combination therapy, noting that AFP or DCP response at 6–8 weeks post-treatment serves as an early predictor of superior oncological outcomes. Shang et al.'s retrospective analysis revealed that triple combination immunotherapy confers significantly enhanced survival benefits over standard chemotherapy as second-line treatment for advanced biliary tract cancer. In a multicenter cohort study, Zhao et al. delineates the distinct advantages of transarterial chemoembolization combined with lenvatinib plus tislelizumab for unresectable HCC. Gkika et al., through a pooled analysis of two prospective studies, elucidates the prognostic role of the ALBI score in patients receiving stereotactic body radiotherapy for locally advanced primary liver tumors.

Citrate synthase (CS), a key rate-limiting enzyme in the tricarboxylic acid (TCA) cycle, plays a crucial role in cancer progression, though its mechanism in promoting liver cancer growth remains incompletely understood. Recent evidence indicates that succinylation of CS is essential for sustaining mitochondrial function and driving cellular proliferation in liver cancer cells. Cao et al. demonstrated that targeting SIRT5-mediated de-succinylation of CS represents a promising therapeutic strategy for hepatocellular carcinoma. Their work revealed that SIRT5 interacts directly with CS to mediate de-succinylation specifically at lysine 375 (K375). Succinylation at CS-K375 was shown to enhance mitochondrial activity and ATP production in HepG2 cells while reducing intracellular reactive oxygen species (ROS) levels and promoting proliferation. Conversely, de-succinylation at K375 significantly impaired mitochondrial function, decreased ATP levels, elevated ROS accumulation, and induced apoptosis in HepG2 cells. These findings not only elucidate the regulatory mechanism of the SIRT5/citrate synthase signaling axis but also offer novel therapeutic insights for HCC treatment.

A well-conducted literature review holds critical importance, enabling researchers to rapidly comprehend the fundamental landscape and research advancements within a field. Gan et al. provide a comprehensive analysis of the dual role of Nrf2 signaling in hepatocellular carcinoma, encompassing its contributions to tumor development, immune evasion mechanisms, and associated therapeutic challenges. Similarly, Tian et al. offer an extensive examination of non-coding RNA regulatory mechanisms in hepatocellular carcinoma, highlighting their implications for both therapeutic strategies and prognostic assessment.

We hope that the findings from the papers published in this Research Topic can have a substantial impact on real-world clinical practice. We also expect to initiate Volume IV of the Research Topic in the near future and receive more interesting and valuable papers.
